# Ahmed to Baerveldt glaucoma drainage device exchange in pediatric patients

**DOI:** 10.1186/s12886-023-03074-1

**Published:** 2023-07-11

**Authors:** Adam Jacobson, Brenda L. Bohnsack

**Affiliations:** 1 Department of Ophthalmology and Visual Sciences, University of Michigan, 1000 Wall Street, Ann Arbor, MI 48105 USA; 2 Department of Ophthalmology, Northwestern University Feinberg School of Medicine, 645 N. Michigan Ave, Chicago, IL 60611 USA; 3 Division of Ophthalmology, Ann & Robert H. Lurie Children’s Hospital of Chicago, Box 70, 225 E. Chicago Ave, Chicago, IL 60611 USA

**Keywords:** Childhood glaucoma, Glaucoma drainage device, Ahmed implant, Baerveldt implant

## Abstract

**Background:**

There is no consensus and few reports as to the surgical management of encapsulated Ahmed glaucoma drainage devices (GDD) which no longer control intraocular pressure (IOP), especially within the pediatric population. The purpose of this study was to report outcomes of exchanging the Ahmed GDD for a Baerveldt GDD in children with refractory glaucoma.

**Methods:**

Retrospective review of children (< 18yrs) who underwent removal of Ahmed FP7 and placement of Baerveldt 350 (2016–2021) with ≥ 3-month follow-up. Surgical success was defined as IOP 5–20 mmHg without additional IOP-lowering surgeries or visually devastating complications. Outcomes included change in best-corrected visual acuity (BCVA), intraocular pressure (IOP), and number of glaucoma medications.

**Results:**

Twelve eyes of 10 patients underwent superotemporal Ahmed FP7 to Baerveldt 350 GDD exchange at 8.8 ± 3.6 years. Time to Ahmed failure was 2.7 ± 1.9 years with 1-, 3-, and 5-year survival rates of 83% with a 95% CI[48,95], 33% with a 95% CI[10, 59], and 8% with a 95% CI[0, 30]. At final follow-up (2.5 ± 1.8 years), success rate for Baerveldt 350 GDDs was 75% (9 of 12 eyes) with 1 and 3-yr survival rates of 100% and 71% with 95% CI[25,92], respectively. IOP (24.1 ± 2.9 vs. 14.9 ± 3.1 mmHg) and number of glaucoma medications (3.7 ± 0.7 vs. 2.7 ± 1.1) were significantly decreased (*p* < 0.004). BCVA remained stable. Two eyes required cycloablation and 1 eye developed a retinal detachment.

**Conclusions:**

Ahmed removal with Baerveldt placement can improve IOP control with fewer medications in cases of refractory pediatric glaucoma. However, more eyes with greater follow-up are required to determine long-term outcomes.

## Precis

Exchange of a failed Ahmed FP7 implant for a Baerveldt 350 glaucoma drainage device can improve IOP control with fewer glaucoma medications in cases of refractory pediatric glaucoma.

## Background

Glaucoma drainage devices (GDDs) are often used in cases of pediatric glaucoma to decrease intraocular pressure (IOP) and preserve vision [1–5]. The valved Ahmed and non-valved Baerveldt implants are most popular amongst pediatric and glaucoma specialists. While the valved Ahmed implant has the benefits of immediately lowering IOP and less risk of hypotony, early plate encapsulation resulting in the hypertensive phase can lead to failure [6, 7]. In contrast, the non-valved Baerveldt GDD may have greater long-term success, but carries with it a higher risk of postoperative hypotony [8, 9]. As a result, the valved Ahmed implant is often used as a first-line GDD, especially by surgeons not familiar or comfortable with managing a non-valved implant.

In refractory cases of glaucoma, elevated IOP due to failure of a valved Ahmed implant frequently requires additional surgery [10]. Options include implant revision with removal of the capsule, placement of a second GDD in a different quadrant, and cycloablation [11–13]. In addition, Ahmed implants, especially those in the superotemporal quadrant, can be removed and replaced with a Baerveldt GDD [14]. However, there is a paucity of information regarding outcomes of this procedure, especially in pediatric cases. In the current study, we present outcomes of a cohort of children who underwent exchange of a valved Ahmed FP7 implant for a Baerveldt 101–350 (BV350) GDD.

## Methods

A retrospective case series identified patients 18 years of age or younger who underwent explantation of a failed valved Ahmed FP7 GDD (New World Medical, Rancho Cucamonga, CA) with concurrent placement of a BV350 GDD (Abbott Medical Optics, Santa Ana, CA) at the University of Michigan or the Ann & Robert H. Lurie Children’s Hospital of Chicago between 01/2016 and 01/2022 by a single surgeon (BLB). The use of Ahmed FP7 and BV350 GDDs are not FDA approved for use in children, but are commonly used in clinical practice [1–5]. Exclusion criteria included less than 3 months of post-operative follow-up or surgery performed by other surgeons. This study was declared exempt, without need for patient approval or informed consent, due to its retrospective nature by the Institutional Review Boards of the University of Michigan and the Ann & Robert H. Lurie Children’s Hospital of Chicago. The study adhered to the tenets of the Declaration of Helsinki. Data collection was de-identified and HIPAA compliant.

Data collected included ocular diagnoses, age at glaucoma surgeries, surgical details, and complications. Childhood glaucomas were diagnosed and classified based on the World Glaucoma Association consensus [15]. Preoperative exam was defined as the examination immediately prior to Ahmed removal and BV350 placement. Examination details obtained included best corrected visual acuity (BCVA), intraocular pressure (IOP), and number of glaucoma medications (oral and topical). IOP was measured by Icare (Revenio, Vantaa, Finland), Tono-pen (Reichert, Depew, NY), or Goldmann applanation. GDD success was defined as IOP between 5 and 21 mmHg (± glaucoma medications), no visually devastating complications, or no additional IOP-related surgery. The secondary outcomes were change in BCVA, IOP, and number of glaucoma medications at final follow-up. Datasets used and analyzed during the current study are available from the corresponding author on reasonable request.

For the surgical procedure, a 4–0 or 5–0 polypropylene suture was placed retrograde from the BV350 plate into the tube. The tube was then ligated with a 6–0 polyglactin suture. A conjunctival incision was created approximately 8 mm posterior to the superotemporal limbus. Dissection was carried through Tenons capsule and scar tissue until the anterior plate and tube of the Ahmed implant was identified. If a patch graft was present, the plane between the graft and sclera was identified and the graft was carefully dissected off of the underlying tissues to expose the tube anteriorly to the sclerostomy. Often the anterior aspect of the graft was left attached to the sclera and could be reused. The capsule surrounding the tube was incised and a 6–0 polyglactin suture was used to ligate the tube half way between the sclerostomy and the plate. The tube was cut just anterior to the plate, leaving the ligated portion of the tube within the eye. The capsule around the plate was carefully dissected from the overlying Tenons capsule. The posts within the fixation holes of the plate were incised to release the plate from the capsule and the plate was removed from the field. The capsule was then dissected from the sclera and removed. The lateral and superior rectus muscles were isolated on muscle hooks. The plate of the prepared BV350 GDD was placed posterior to the lateral and superior rectus muscle insertions and the plate was secured to the sclera with 8–0 nylon sutures approximately 8 mm posterior to the limbus. The BV350 tube was trimmed to an appropriate length. The cut end of the Ahmed tube was then removed and the Baerveldt tube was placed through the same sclerostomy. The tube was secured to the sclera with 9–0 nylon and then covered either with the previous or a new scleral patch graft. The Tenons capsule and conjunctiva were then closed in a double layered fashion with 8–0 polyglactin.

Statistical analyses included Wilcoxon Rank Sum test (for comparisons between preoperative and postoperative values), Kaplan–Meier survival curves with Log-rank (Mantel-Cox) test with 95% confidence intervals (CI), and non-linear regression analysis. Tests were performed with GraphPad Prism 9.0 (GraphPad, La Jolla, CA). All tests were 2-sided with *p*-values less than 0.05 considered statistically significant.

## Results

Twelve eyes of ten patients (4 male:10 female) underwent explantation of an Ahmed FP7 GDD with simultaneous implantation of a BV350 GDD at an average age of 8.8 ± 3.6 years (median 8.3, range 2.6–13.9). Six patients were Caucasian, one of who identified as Hispanic, and 4 were Black (Table 1). Diagnoses included primary congenital glaucoma (3 eyes of 2 patients), glaucoma following cataract surgery (3 eyes of 3 patients), and glaucoma secondary to non-acquired ocular anomalies (6 eyes of 5 patients).

**Table 1:** Demographics of eyes of patients that underwent an Ahmed to Baerveldt exchange

Pt #	Diagnosis	Eye	Gender	Race/Ethnicity
1	PCG	OS	Female	Caucasian/Hispanic
2	PCG	OD OS	Female	Black
3	GFCS	OD	Female	Caucasian
4	GFCS	OS	Male	Caucasian
5	GCFS	OD	Male	Caucasian
6	ARS	OD OS	Male	Black
7	ARS	OS	Female	Caucasian
8	ARS	OS	Female	Caucasian
9	ARS	OS	Male	Black
10	JXG	OD	Female	Black

*Abbreviations:* *OD* Right, *OS* Left, *PCG* Primary congenital glaucoma, *GFCS* Glaucoma following cataract surgery, *ARS* Axenfeld-Rieger syndrome, *JXG* Juvenile xanthogranuloma

Six eyes of five patients had previously undergone between 1 and 3 angle surgeries (goniotomy or trabeculotomy) prior to Ahmed FP7 placement (Table 2, 1.7 ± 0.8 surgeries). Only the 3 eyes with glaucoma following cataract surgery underwent other non-glaucoma related intraocular surgeries including lensectomy (3 eyes of 3 patients), secondary intraocular lens placement (1 eye) and pupilloplasty (2 eyes of 2 patients).

**Table 2:** Surgical details for each eye including prior prior surgeries and length of success of Ahmed and Baerveldt GDDs

Pt #	Eye	# Angle Surgeries Prior to Ahmed FP7	# of Non-Glaucoma Intraocular Surgeries Prior to Ahmed	Age at Ahmed FP7 (yrs)	Length of Ahmed Success (yrs)	# Glaucoma Surgeries Between Ahmed FP7 and Baerveldt 350	Age at Baerveldt 350 placement (yrs)	Length of time between Ahmed and Baerveldt surgeries (yrs)	Baerveldt success at Final Follow-up	Length of Baerveldt Success (yrs)
1	OS	2	0	0.43	2.20	0	2.63	2.20	Yes	1.88
2	OD	1	0	0.33	1.67	2 Inferotemporal Ahmed FP8 Transcleral cycloablation	8.35	8.01	Yes	3.50
2	OS	2	0	0.25	3.75	2 Inferotemporal Ahmed FP8 Transcleral cycloablation	8.29	8.04	Yes	3.56
3	OD	0	2	7.35	2.43	0	9.78	2.43	Yes	1.31
4	OS	1	3	4.90	1.91	0	6.80	1.91	Yes	0.99
5	OD	3	1	0.71	5.96	1 Tube revision to trim tube length^a^	6.67	5.96	Yes	0.86
6	OD	1	0	10.74	0.73	4 Tube revision due to iris occlusion × 2 Transcleral cycloablation Inferotemporal Ahmed FP8	13.12	2.38	No	2.47
6	OS	0	0	11.60	0.44	5 Transcleral cycloablation × 2 Inferonasal Baerveldt 250 Trabeculectomy with Mitomycin C Trabeculectomy revision due to failure	13.94	2.34	Yes	4.81
7	OS	0	0	1.88	1.99	1 Inferonasal Baerveldt 250	4.49	2.61	No	3.99
8	OS	0	0	5.24	6.02	0	11.25	6.02	Yes	1.03
9	OS	0	0	5.24	2.51	2 Inferotemporal Ahmed FP8 Tube revision to lengthen tube due to retraction	11.70	6.46	Yes	2.98
10	OD	0	0	0.47	4.12	0	4.59	4.12	No	2.46

*Abbreviations:* *OD* Right, *OS* Left

^a^Tube trimming surgery not done for IOP control, but to decrease risk of tube-corneal touch

**Fig. 1:**
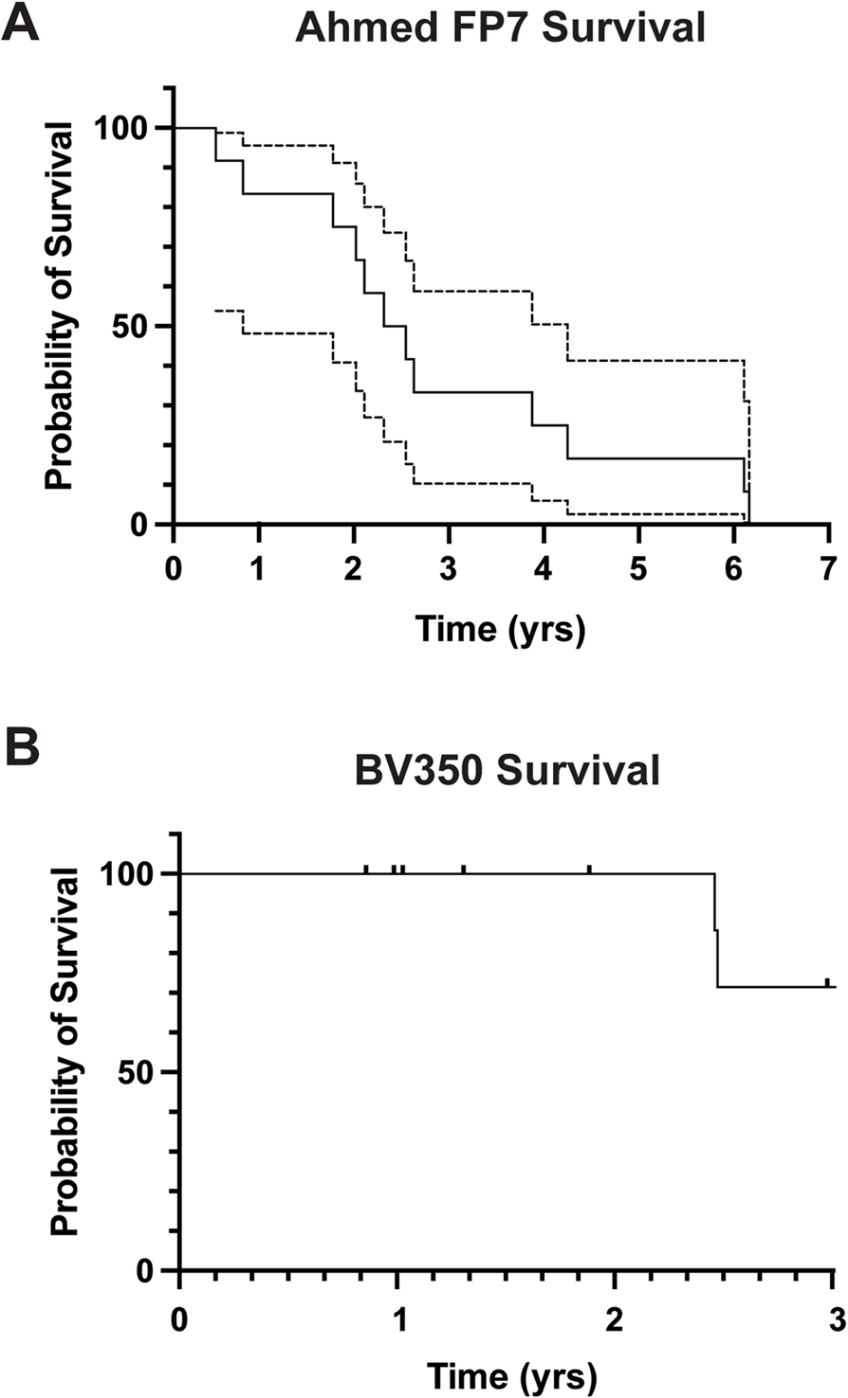
Survival rates of GDD devices. **A** Kaplan–Meier survival analysis of Ahmed FP7 GDDs showed 1-, 3-, and 5-year survival rates of 83% with a 95% CI [48,95], 33% with a 95% CI [10,59], and 8% with a 95% CI [0,30], respectively. **B** At final follow-up, BV350 glaucoma drainage devices showed survival rates of 100% and 71% with 95% CI [25,92], at 1- and 3-years, respectively

All Ahmed FP7 GDDs were implanted in the superotemporal quadrant at an average age of 4.4 ± 4.1 years (Table 2, median 3.4, range 0.3–11.6). The average time to Ahmed failure was 2.7 ± 1.9 years (median 2.3, range 0.4–6.0) with 1-, 3-, and 5-year survival rates of 83% with a 95% CI [48,95], 33% with a 95% CI [10,59], and 8% with a 95% CI [0,30], respectively (Fig. 1A). There was no correlation (R^2^ = 0.04) between number of prior intraocular surgeries and time to Ahmed FP7 failure. Six eyes of 4 patients failed prior to BV350 placement due to intermediate glaucoma surgeries (Table 2) including Ahmed FP7 revision for iris occlusion or tube retraction (2 eyes of 2 patients), transcleral cyclophotocoagulation (4 eyes of 2 patients), inferotemporal Ahmed FP8 GDD placement (4 eyes of 3 patients), inferonasal Baerveldt 250 GDD placement (2 eyes of 2 patients), and trabeculectomy with mitomycin C (1 eye of 1 patient). One eye (patient 5) underwent surgery, unrelated to IOP control, in order to trim the intraocular portion of the Ahmed tube to protect the cornea.

The average length of time between initial Ahmed FP7 placement and GDD exchange was 4.4 ± 2.5 years (median 3.4 years, range 1.9–8.0 years) such that the mean age at the time of BV350 placement was 8.8 ± 3.6 years (median 8.3, range 2.6–13.9 years). At final follow-up (2.5 ± 1.3 years, median 2.5, range 0.9–5.6), the BV350 success rate was 75% (9 of 12 eyes). Survival analysis (Fig. 1B) showed 1- and 3-yr survival rates of 100% and 71% with 95% CI[25,92]. As with Ahmed GDDs, there was no correlation (R^2^ = 0.1) between the number of prior intraocular surgeries and success time of BV350. When the eyes were separated into 2 groups based on whether IOP-related surgeries were performed between Ahmed GDD placement and GDD exchange, the 6 eyes that did not require any IOP-related surgery had significantly shorter follow-up (1.4 ± 0.6 years, range 0.9–2.5 years, *p* < 0.04) compared to the 6 eyes which underwent at least 1 IOP-related surgery (e.g. transcleral cyclophotocoagulation, second GDD, or trabeculectomy with mitomycin C, 3.1 ± 1.6 years, range 2.5–4.8 years). In the group without intervening surgeries the 1-year survival rate was 100% and the success rate at final follow-up was 83%. As none of the eyes had 3 years of follow-up, additional survival rates were not able to be determined. In eyes which underwent surgeries between the Ahmed placement and GDD exchange, the 1- and 3-year survival rates were 100% and 83% with a 95% CI [27,97], respectively and the success at final follow-up was 67%.

At final follow-up, 3 eyes of 3 patients were deemed failures. Two eyes of 2 patients with Axenfeld Rieger Syndrome required transcleral cycloablation for plate encapsulation leading to elevated IOP after 2.47 years (patient 6, right eye) and 3.99 years (patient 7, left eye). One patient with JXG (patient 8) was lost to follow up for 2 years after the GDD exchange surgery, and returned with a chronic funnel retinal detachment and dislocation of the crystalline lens into the vitreous. Additional surgery was unsuccessful and the eye eventually became phthisical.

At final follow-up, IOP was significantly decreased (*p* < 0.001, Table 3) compared to the examination immediately prior to GDD exchange (15.2 ± 4.5 mmHg vs. 24.1 ± 2.9 mmHg). Similarly, the number of glaucoma medications was also decreased (*p* < 0.004) at final follow-up (2.7 ± 1.1 vs. 3.7 ± 0.7). Further, there was no significant difference (*p* = 0.39) in LogMar BCVA between the preoperative (0.8 ± 0.6) and final examination (0.8 ± 0.6).

**Table 3:** Preoperative and postoperative examination details

**Pt #**	**Eye**	**Preoperative BCVA (Logmar)**	Preoperative **IOP**	**Preoperative Glaucoma Medications**	**Final BCVA**	**Final IOP**	**Final Glaucoma Medications**	**Follow-up Time (yrs)**
1	OS	1.28	24	3	0.88	19	2	1.88
2	OD	1.40	20	4	1.3	13	3	3.50
2	OS	0.48	28	4	0.4	15	3	3.56
3	OD	0.54	25	4	0.7	14	2	1.31
4	OS	1.18	26	3	1.7	18	2	0.99
5	OD	1.90	27	3	1.9	20	0	0.86
6	OD	0.54	23	4	0.54	16	3	5.63
6	OS	0.60	19	4	0.48	20	3	4.81
7	OS	Right Eye Preference	25	3	0.50	10	3	4.07
8	OS	0.10	21	4	0.18	11	4	1.03
9	OS	0.40	24	5	0.48	20	4	2.98
10	OD	1.90	25	3	2.80	6	3	2.46

*Abbreviations:* *OD* Right, *OS* Left, *BCVA* best corrected visual acuity, *IOP* intraocular pressure

## Discussion

GDDs are an important mainstay of treating childhood glaucomas and both Ahmed and Baerveldt implants decrease IOP and the number of glaucoma medications in children [3, 5, 16–19]. The valved Ahmed GDD tends to be more popular due to the immediate IOP lowering effect and decreased risk of post-operative hypotony compared to the non-valved Baerveldt implants. While Ahmed GDDs in children have a greater than 80% success rate at 1 year, by 5-years, success is often less than 50% [3, 16, 17]. Failure of Ahmed implants is most often due to plate encapsulation, which is associated with a robust and early hypertensive phase [3, 16, 17]. A challenge is determining next surgical steps after Ahmed implant failure. In the current study, we present a cohort of pediatric patients who underwent removal of an encapsulated Ahmed FP7 with placement of a BV350 GDD.

The strategy underlying this GDD exchange is that Baerveldt implants as non-valved devices show greater longevity and pressure lowering effect due to less plate encapsulation and larger available plate sizes [8, 9, 18]. Early postoperative capsule formation around a non-valved implant occurs in the absence of pro-inflammatory factors within aqueous humor that are hypothesized to accelerate encapsulation of a valved device. Further, most surgeons place the initial Ahmed GDD in the superotemporal quadrant, which is also the preferred location for the Baerveldt 350 implant given the size of the plate and resulting bleb. This GDD exchange surgery leaves other quadrants of the globe untouched if additional glaucoma surgeries are required. Our study was a small cohort of 12 eyes and the average length of follow-up was only 2.5 years. However, to the best of our knowledge, there has only been one other study with 9 eyes of adult patients that reported outcomes of this procedure with an average follow-up of 3.9 years [14]. In Zuo and Lesk, the majority of the Ahmed implants were the S2 model (7 of 9 eyes), which is the original valve made of polypropylene, not the FP7, which is silicone [14]. Nonetheless, studies have shown that there is no significant difference in IOP-lowering effect or success rate between these two models [20]. The success rate based on their criteria (IOP of 21 mmHg or lower and a 20% IOP reduction from baseline starting two months post-operatively) at final follow-up was 67% (7 of 9 eyes). Importantly, 2 additional patients, who were not considered failures by Zuo and Lesk’s criteria, required IOP-lowering surgery (orphan trabeculectomy and tube revision for occlusion) within the first two post-operative months [14]. Two of the failures in the current study required cycloablation approximately 2.5 and 4 years after the GDD exchange, and at final follow-up had achieved IOP control. One patient, who was lost to follow-up, returned with a chronic unrepairable retinal detachment. This eye was considered a failure, although given the lack of intermediate follow-up, it is unclear the etiology of the retinal detachment and its relation to the GDD exchange surgery.

There are additional surgical options for encapsulated Ahmed GDDs. The Ahmed implant can be revised to remove the dense capsule that surrounds the plate [11]. While this may yield temporary IOP control, the plate often becomes re-encapsulated leading to recurrent elevated IOP. The use of mitomycin C in conjunction with capsule removal showed a success rate of 58% at 3 years, but increased rate of erosion requiring device explantation [13]. Placement of a second GDD in a different quadrant is a common choice. Studies have demonstrated that implantation of a second Ahmed GDD shows success rates of 60–87% at 12–18 months, but decreases to 52% at 3 years. Further, the most common complication was corneal edema or bullous keratopathy in 13–25% of the patients [21–23]. A multi-center randomized trial of second aqueous shunts compared to transcleral cyclophotocoagulation showed similar 1-year success rates for both procedures (79% vs. 88%) in 42 eyes of adult patients [12]. However, in this study there was no data regarding the primary GDD type and the secondary implant was a Baerveldt. In addition to corneal decompensation, there are other potential complications related to the location of the second tube. The inferotemporal quadrant is more prone to exposure and can create noticeable blebs under the thin skin of the lower eyelid. Further, GDDs in the superonasal and inferonasal quadrants may interfere with the superior and inferior oblique muscles, respectively, leading to challenging vertical or torsional strabismus [24].

The exchange of an Ahmed GDD for a BV350 implant requires removal of the device as well as the capsule which is often adhered to both the sclera and overlying Tenons capsule. A concern regarding the exchange surgery is that scarring from the previous Ahmed may influence wound healing such that the Baerveldt plate may more quickly encapsulate leading to elevated IOP. In the current study, the 1- and 3- year survival rates of the Baerveldt 350 GDDs were 100% and 71%. This is similar to other studies not involving a GDD exchange that showed Baerveldt success rates of 80–90% at 1-year and 60–70% by 5–6 years [18, 25]. The most common cause of Baerveldt failure is plate encapsulation that increases resistance to outflow. Like Ahmed implants, Baerveldt devices can be revised or a second GDD can be placed in a different quadrant. However, neither of these approaches may yield long-term IOP control and potentially increase scarring and utilize additional quadrants that may limit future surgical options. As a result, at least in our practice cycloablation, either transcleral or endoscopic, is often employed if IOP is increased due to plate encapsulation. This approach does rely on some amount of outflow through the capsule and may not be effective if there is dense encapsulation. Further, cycloablation can be less predictable and there is a risk of chronic hypotony and subsequent phthisis with over treatment [12].

In cases of refractory glaucoma, the goal is to obtain IOP control while maintaining vision. In our cohort, there was no significant difference between preoperative and final BCVA, although the average LogMar visual acuity was 0.8 (~ 20/120). The lower visual acuity in these eyes can be attributed to many factors including glaucomatous optic neuropathy, corneal damage, high refractive error, and amblyopia. In the patient who developed a retinal detachment, she was densely amblyopic in her affected eye and although her visual acuity decreased from count fingers to light perception, there was no effect on her overall visual function as her unaffected eye was 20/20. Further, Like Zuo and Lusk, exchange of an Ahmed GDD to a BV350, IOP and the number of glaucoma medications were significantly decreased at final follow-up [14].

There are limitations to this study, which include the small sample size with variable follow-up times and the retrospective nature of this study. Further, this cohort represents a heterogenous sample of childhood glaucomas and different number of prior glaucoma surgeries which may influence the success of the procedure. Despite these limitations, it is important to recognize that in the situation of failed Ahmed GDD, this surgical procedure is a plausible option for obtaining IOP control with fewer medications and importantly preserving the conjunctiva in other quadrants. This is the first report of exchange of an Ahmed implant for a BV350 GDD in the pediatric population and additional studies are required to better evaluate longer term outcomes with a greater number of eyes.

## Conclusions

In pediatric patients with refractory glaucoma, same quadrant exchange of a valved Ahmed GDD for a non-valved Baerveldt can improve IOP control with fewer medications at final follow-up. While more eyes with greater follow-up are required, this surgical procedure is a viable option for encapsulated Ahmed GDDs which no longer adequately control IOP in children.

## Data Availability

The datasets used and analyzed during the current study are available from the corresponding author on reasonable request.

## Abbreviations

IOP: Intraocular pressure
GDD: Glaucoma drainage device
BV350: Baerveldt 101–350 glaucoma drainage device
CI: Confidence interval

## References

[1] Netland PA, Walton DS (1993). Glaucoma drainage implants in pediatric patients. Ophthalmic Surg.

[2] Waheed S, Ritterband DC, Greenfield DS, Liebmann JM, Sidoti PA, Ritch R (1997). Bleb-related ocular infection in children after trabeculectomy with mitomycin C. Ophthalmology.

[3] Eksioglu U, Yakin M, Sungur G, Satana B, Demirok G, Balta O, Ornek F (2017). Short- to long-term results of Ahmed glaucoma valve in the management of elevated intraocular pressure in patients with pediatric uveitis. Can J Ophthalmol.

[4] Razeghinejad MR, Havens SJ, Katz LJ (2017). Trabeculectomy bleb-associated infections. Surv Ophthalmol.

[5] Medert CM, Cavuoto KM, Vanner EA, Grajewski AL, Chang TC (2021). Risk factors for glaucoma drainage device failure and complication in the pediatric population. Ophthalmology Glaucoma.

[6] Nouri-Mahdavi K, Caprioli J (2003). Evaluation of the hypertensive phase after insertion of the Ahmed Glaucoma Valve. Am J Ophthalmol.

[7] Jung KI, Park H, Jung Y, Park CK (2015). Serial changes in the bleb wall after glaucoma drainage implant surgery: characteristics during the hypertensive phase. Acta Ophthalmol.

[8] Tsai JC, Johnson CC, Kammer JA, Dietrich MS (2006). The Ahmed shunt versus the Baerveldt shunt for refractory glaucoma II: longer-term outcomes from a single surgeon. Ophthalmology.

[9] Christakis PG, Zhang D, Budenz DL, Barton K, Tsai JC, Ahmed IIK (2017). Groups A-AS: Five-year pooled data analysis of the Ahmed Baerveldt comparison study and the Ahmed versus Baerveldt study. Am J Ophthalmol.

[10] Lavin MJ, Franks WA, Wormald RP, Hitchings RA (1992). Clinical risk factors for failure in glaucoma tube surgery. A comparison of three tube designs. Arch Ophthalmol.

[11] Shah AA, WuDunn D, Cantor LB (2000). Shunt revision versus additional tube shunt implantation after failed tube shunt surgery in refractory glaucoma. Am J Ophthalmol.

[12] Shaefer JL, Levine MA, Martorana G, Koenigsman H, Fran Smith M, Sherwood MB (2015). Failed glaucoma drainage implant: long-term outcomes of a second glaucoma drainage device versus cyclophotocoagulation. Br J Ophthalmol.

[13] Salimi A, N. K, Harasymowycz PJ: Tube shunt revision with excision of fibrotic capsule using mitomycin C with and without ologen-a collagen matrix implant: a 3-year follow-up study. J Glaucoma 2019, 28(11):989–996. 10.1097/IJG.0000000000001371 31567908

[14] Zuo W, Lesk MR (2018). Surgical outcome of replacing a failed Ahmed glaucoma valve by a Baerveldt glaucoma implant in the same quadrant in refractory glaucoma. J Glaucoma.

[15] Weinreb RN, Grajewski AL, Papadopoulos M, Grigg JR, Freedman SF: Childhood Glaucoma. The 9th Consensus Report of the World Glaucoma Association. The Netherlands: Kugler Publications; 2013.

[16] Pakravan M, Esfandiari H, Yazdani S, Doozandeh A, Dastborhan Z, Gerami E, Kheiri B, Pakravan P, Yaseri M, Hassanpour K (2019). Clinical outcomes of Ahmed glaucoma valve implantation in pediatric glaucoma. Eur J Ophthalmol.

[17] Spiess K, Calvo JP: Outcomes of Ahmed glaucoma valve in paediatric glaucoma following congenital cataract surgery in persistent foetal vasculature. Eur J Ophthalmol 2020, 1120672120919066. 10.1177/1120672120919066 32354227

[18] Jacobson A, Besirli CG, Bohnsack BL: Outcomes of Baerveldt glaucoma drainage devices in pediatric eyes. J Glaucoma 2021, Dec 21. 10.1097/IJG.0000000000001970 PMC9148673 34930874

[19] Jacobson A, Bohnsack BL (2022). Ologen augmentation of Ahmed valves in pediatric glaucomas. J AAPOS.

[20] Elbaklish KH, Gomaa WA (2020). A one-year follow-up of two Ahmed glaucoma valve models (S2 and FP7) for refractory glaucoma: A prospective randomized trial. Clin Ophthalmol.

[21] Jiménez-Roman J, Gil-Carrasco F, Costa VP, Schimit RB, Lerner F, Santana PR, Vascocellos JP, Castillejos-Chévez A, Turati M, Fabre-Miranda K (2016). Intraocular pressure control after the implantation of a second Ahmed glaucoma valve. Int Ophthalmol.

[22] Ko SJ, Hwang YH, Ahn SI, Kim HK (2016). Surgical outcomes of additional Ahmed glaucoma valve implantation in refractory glaucom. J Glaucoma.

[23] Becerril-Cazadero R, Seibold LK, Turati-Acosta M, Jiménez-Roman J, Fabre-Miranda K, Han Y, Lazcano-Gomez G (2020). Surgical outcomes of a second Ahmed glaucoma valve implant for the treatment of refractory glaucoma. J Glaucoma.

[24] Al-Lozi A, Umfress AC, Stinnett SS, Freedman SF (2022). Outcomes of inferonasal galucoma drainage device surgery in the management of childhood glaucoma. J AAPOS.

[25] Autrata R, Helmanova I, Oslejskova H, Vondracek P, Rehurek J (2007). Glaucoma drainage implants in the treatment of refractory glaucoma in pediatric patients. Eur J Ophthalmol.

